# Molecular epidemiological study of feline coronavirus strains in Japan using RT-PCR targeting nsp14 gene

**DOI:** 10.1186/s12917-015-0372-2

**Published:** 2015-03-11

**Authors:** Yoshikazu Tanaka, Takashi Sasaki, Ryo Matsuda, Yosuke Uematsu, Tomohiro Yamaguchi

**Affiliations:** Department of Veterinary Hygiene, Veterinary School, Nippon Veterinary & Life Science University, 1-7-1 Kyounan, Musashino, Tokyo 180-8602 Japan; Canine Lab., Inc., Nokodai-Tamakoganei Venture Port 302, 2-24-16, Koganei, Tokyo 184-0012 Japan

**Keywords:** Feline coronavirus, Epidemiology, Phylogenetic study

## Abstract

**Background:**

Feline infectious peritonitis is a fatal disease of cats caused by infection with feline coronavirus (FCoV). For detecting or genotyping of FCoV, some RT-PCR plus nested PCR techniques have been reported previously. However, referring to the whole genome sequences (WGSs) registered at NCBI, there are no detection methods that can tolerate the genetic diversity among FCoV population. In addition, the quasispecies nature of FCoV, which consists of heterogeneous variants, has been also demonstrated; thus, a universal method for heteropopulations of FCoV variants in clinical specimens is desirable.

**Results:**

To develop an RT-PCR method for detection and genotyping of FCoV, we performed comparative genome analysis using WGSs of 32 FCoV, 7 CCoV and 5 TGEV strains obtained from NCBI. As the PCR target, we focused on the nsp14 coding region, which is highly conserved and phylogenetically informative, and developed a PCR method targeting nsp14 partial sequences. Among 103 ascites, 45 pleural effusion and 214 blood specimens from clinically ill cats, we could detect FCoV in 55 (53.4%), 14 (31.1%) and 19 (8.9%) specimens using the present method. Direct sequencing of PCR products and phylogenetic analysis allowed discrimination between type I- and II-FCoV serotypes. Our nsp14 amino acid sequence typing (nsp14 aa ST) showed that the FCoV clone with sequence type (ST) 42, which was the most predominant genotype of WGS strains, was prevalent in domestic cats in Japan.

**Conclusions:**

Our nsp14 PCR scheme will contribute to virus detection, epidemiology and ecology of FCoV strains.

**Electronic supplementary material:**

The online version of this article (doi:10.1186/s12917-015-0372-2) contains supplementary material, which is available to authorized users.

## Background

Feline coronavirus (FCoV) can be classified into two biotypes, namely low virulent feline enteric coronavirus (FECV) and highly virulent feline infectious peritonitis virus (FIPV) [1]. Clinical appearance of FECV, if any, is characterized by mild enteritis. In contrast, FIPV efficiently replicates in macrophages/monocytes, and can lead to FIP, which is a highly lethal systemic granulomatous disease [2]. FIPV exists in two serotypes based on virus neutralizing antibodies, type I and type II [1,3]. Serotype I virus has a distinctive spike protein, while the spike protein of serotype II is a recombinant protein between feline and canine enteric coronaviruses [4]. Type I FECVs/FIPVs predominate throughout the world, but type II strains appear to be more adaptable to tissue culture. However, type I strains are more likely to cause clinical FIP signs [3]. FCoV is also common in healthy cats worldwide; less than 10% of FCoV seropositive cats develop FIP [5-9]. Therefore, measuring antibody levels against FCoV is rarely of diagnostic value in FIP. Thus, Histopathological examination of infected tissues is needed for the aetiological diagnosis of FIP [10,11].

RT-PCR plus nested PCR and real-time PCR techniques have allowed the detection or genotyping of FCoV [3,12-15]. However, they have not been fully reevaluated whether they can tolerate the genetic diversity of FCoV. In recent years, whole genome sequences of clinical strains of FCoV have been registered in the NCBI database by Rottier et al. at the J. Craig Venter Institute, and we have been able to readily obtain nucleotide sequences of the FCoV genome. With regard to genome information, there have been no reports on PCR-based methods that can tolerate the genetic diversity in whole genome sequenced FCoV strains. Thus, a detection method for all variants of FCoV is highly desirable.

In the present study, in order to construct a universal method for FCoV variants, we performed a genome-wide analysis of FCoV, and developed an RT-PCR method for detecting FCoV in clinical specimens. Consequently, direct sequencing of PCR products and phylogenetic analysis allowed discrimination between type I and II serotypes of FCoV. Using this method, we investigated the population genetics of FCoV strains from diseased cats in Japan.

## Methods

### Bioinformatics for FCoV, CCoV and TGEV

As shown in Additional file 1, thirty-two FCoV strains, seven canine coronavirus (CCoV) strains, four transmissible gastroenteritis coronavirus (TGEV) strains, a porcine respiratory coronavirus (PRCV) strain and a Mink coronavirus (MiCoV) strain were used for comparative genome analysis. All sequences were obtained from the NCBI database. Gene searches and annotation were carried out by GeneMarkS (http://opal.biology.gatech.edu/genemarks.cgi) [16], RAST annotation server (http://rast.nmpdr.org) [17] and the blast-based method. MAFFT v7.037 was used for multiple sequence alignment [18], and Aminosan v1.0.2011 was performed for amino-acid substitution model selection [19]. Phylogenetic inference using maximum likelihood (ML) and bootstrapping was performed using MEGA v5.05 [20].

In order to perform a genome-wide comparison among type I- and II-FCoV, CCoV and TGEV strains, we used the Genotyping tool at NCBI (http://www.ncbi.nlm.nih.gov/projects/genotyping/formpage.cgi), which helps identify genotype and recombinant sequences using the blast-based method [21]. Default values were used; 300 for “window” and 100 for “increment”.

### Cell culture and virus

*Felis catus* whole fetus-4 (fcwf-4; American Type Culture Collection, VA, USA) cells were maintained in Dulbecco’s modified Eagle’s medium (D-MEM, Sigma-Aldrich, Tokyo, Japan) supplemented with 10% fetal bovine serum (JRH, Nissui, Tokyo, Japan). We purified FCoV using linear sucrose gradient ultracentrifugation from FCoV 79–1146 strains (a gift from Tsutomu Hodatsu, Kitasato University, Japan) propagated in fcwf-4 cells.

### RNA extraction and reverse transcription-PCR

Isogen-LS (Nippon Gene, Toyama, Japan) was used for RNA preparation from clinical specimens (whole blood, pleural fluid, ascites, pericardial effusion and cerebral fluid), and fcwf-4 cells infected with FCoV (79–1146 strain) according to the manufacturer’s protocol. Total RNA was reverse-transcribed using the PrimeScript RT-PCR kit (Perfect Real Time; Takara Bio, Shiga, Japan), as reported previously [22].

### Construction of PCR method for detection and genotyping of FCoV strains

In order to construct a PCR method that detects variants in FCoV strains, primers were designed by multiple alignments of nucleotide sequences of the nsp14 genes in all whole genome-sequenced FCoV, closely related subspecies, CCoV and TGEV strains. The primer set nsp14-F (5′-GTGATGCTATCATGACTAG-3′) and nsp14-R (5′-CACCATTACAACCTTCTAA-3′) was used. The expected size of PCR products was 417 bp. The reaction mixture for PCR consisted of 4 μl of cDNA in a total volume of 25 μl composed of 1 U of Ex *Taq* (Takara-Bio), 10 pmol of each primer, 0.2 mM deoxynucleoside triphosphate mixture and 1× reaction buffer (Takara-Bio). Reaction mixtures were thermally cycled once at 95°C for 2 min; 40 times at 95°C for 30 s, 48°C for 35 s, and 72°C for 45 s; and then once at 72°C for 5 min. Using 6 μl of PCR sample, DNA fragments were analyzed by electrophoresis in 1× Tris-acetate-EDTA on a 1% agarose gel stained with ethidium bromide. In addition, these PCR products were directly sequenced using a Big Dye terminator (version 3.1) cycle sequencing kit (Applied Biosystems, Tokyo, Japan) with an ABI Prism 3100 genetic analyzer (Applied Biosystems).

Total RNA was extracted from the fcwf-4 cells infected with FCoV strain 79–1146, and was reverse-transcribed into cDNA. Viral cDNA was quantified using a real-time PCR method, as reported previously [22]. Using cDNA samples of known copy numbers, we evaluated the detection limit of our PCR method targeting nsp14.

### Study population in molecular epidemiological study of FCoV strains from clinically ill cats in Japan

In the period between 2007 and 2014 in Japan, 372 specimens (103 ascites, 45 pleural effusion, 214 blood, 9 cerebral fluid and 1 pericardial effusion), which were obtained in the examination of clinically ill cats for the presence of FCoV, were used in the present study. To detect FCoV in clinical specimens, we performed RT-PCR using a random primer plus a single PCR targeted nsp14 constructed in the present study. To differentiate type I-FCoV from type II-FCoV or CCoV genotypes, direct sequencing of their PCR products and phylogenetic analysis were carried out.

In order to determine the significance of differences in detection rate between ascites, pleural effusion and blood specimens, relative risk and odds ratio were calculated using MedCalc software (http://www.medcalc.org/index.php).

### nsp14 amino acid sequence typing (nsp14 aa ST) and comparison of genetic diversity in FCoV strains between different specimens

In order to devise a genotyping method for FCoV, we assigned an ST number to all distinct nsp14 partial amino acid sequences (135 aa) found in all FCoV strains, which were sequenced in the present study and previously registered in the NCBI database. We compared the population genetic structures of FCoV strains in Japan with whole genome-sequenced strains from 6 different countries.

### Comparison of diversity and evenness indexes of FCoV strains by specimen

The diversity and evenness of ST distribution by specimen type were calculated using Simpson’s diversity index (1-λ) and Pielou’s evenness index (*J’*) [23,24]. These parameters have generally been used for comparisons of biodiversity between geographically or ecologically separated environments. Both values range from 0 (no diversity or evenness) to 1 (extreme diversity or evenness).

## Results

### Genome-wide comparison between FCoV strains and closely related subspecies, CCoV and TGEV

**Figure 1:**
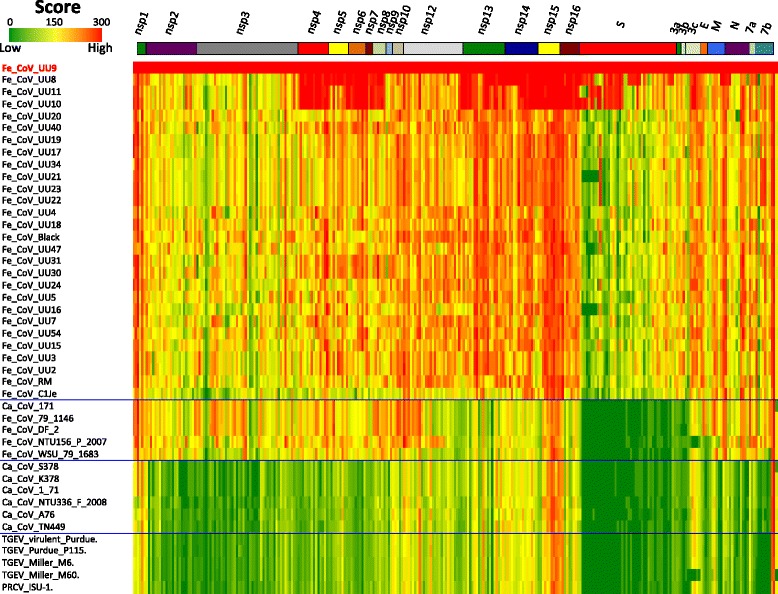
**Genome-wide comparison of FCoV, CCoV, TGEV and PRCV strains registered at NCBI database.** Genomic regions of every 300 bp of strain UU9 were compared with 43 whole genome sequenced strains using BLAST Genotyping tool [21]. The blast score were visualized in a red-yellow-green gradient with green being the top score (300) and red being the bottom score (0), indicating nucleotide similarity against the corresponding region in FCoV strain UU9.

In order to compare the genome structures between type I-FCoV and type II-FCoV, and the closely related subspecies CCoV, TGEV and PRCV, we carried out a blast-based genome-wide comparison. After performing genotyping, genomic regions of 300 bp in each viral strain were color coded according to scores (0 to 300) based on nucleotide similarities against FCoV strain UU9 (Figure 1). In the genome structures of all strains with type II-FCoV genotype, events of large-scale recombination were found in a locus that stretches for about 12,000 bp from the first half of nsp12 to upstream of the nucleocapsid gene, as reported previously [25,26]. The recombination site varied slightly from one strain to another. In the phylogenetic tree based on concatenated sequences of nsp13, nsp14, nsp15, nsp16 and spike, which are protein-coding regions located within recombinant sequences, all type II-FCoV strains were clustered into a clade belonging to canine coronavirus strains (data not shown). Among the alpha-coronavirus 1 subspecies, to which FCoV, CCoV, TGEV and PRCV belonged, the coding regions from nsp14 to nsp16 were the most highly conserved (Figure 1).

### PCR method targeting nsp14 for detection of FCoV strains from clinical specimens

Based on a genome-wide comparison using the Genotyping tool at NCBI, we focused on protein-coding sequences within nsp14 to nsp16 as a candidate PCR target. The sequence region was suited to universal detection of variants because of its highly conserved sequence. Among the primer sets designed in nucleotide sequences conserved in all FCoV, CCoV, TGEV and PRCV strains, we determined the best primer set, nsp14-F and nsp14-R, which allowed amplification of 417 bp of the nsp14 partial sequences. Consequently, this PCR is universally applicable to all alpha-coronavirus 1 subspecies.

**Figure 2:**
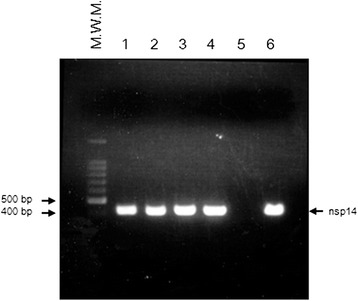
**Agarose gel electrophoresis of nsp14 PCR amplification products from clinical specimens.** Lane 1; pleural fluid, lane 2; ascites, lane 3; pericardial effusion, lane 4; FCoV positive whole blood by a qPCR method, lane 5; FCoV negative whole blood by a qPCR method, lane 6; fcwf-4 cells infected with FCoV strain 79–1146. M.W.M. (molecular weight marker; 100 bp ladder) is loaded on the agarose gel.

We were able to detect FCoV strains from ascites, pleural effusion, pericardial effusion and blood samples using the present method. Few nonspecific bands were found in agarose gel electrophoresis (Figure 2). Specific amplification of target sequences against all PCR products was confirmed by sequencing analysis. To evaluate the detection limit, the present method was applied to cDNA samples of known copy number using PCR. The detection limit was 4.59 × 10^2^ copies/mL.

### Sequence-based differentiation of type I-FCoV from type II-FCoV or CCoV

**Figure 3:**
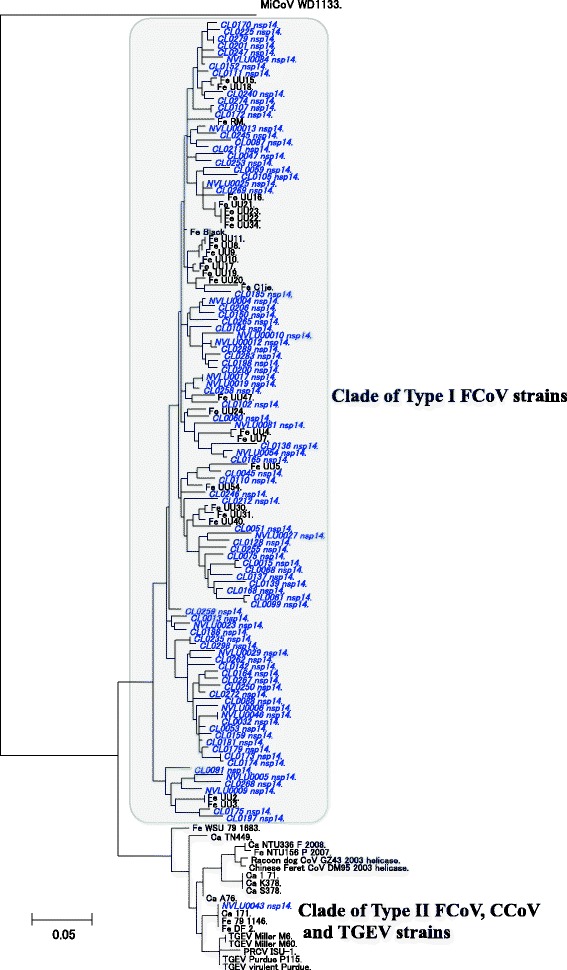
**Phylogenetic tree (ML method) based on nsp14 partial nucleotide sequences (406 bp).** Clinical strains detected in the present study and those obtained from NCBI database were indicated by blue and black letters, respectively.

The nsp14 coding region was located on the recombination hotspot in type II-FCoV, and sequencing analysis of the region permitted discrimination between type I and II serotypes. The nucleotide identity of the nsp14 partial sequence among FCoV, CCoV, TGEV and PRCV strains ranged from 89.9 to 100%. By phylogenetic analysis based on nsp14 partial nucleotide sequences (406 bp), we were able to successfully distinguish the type I-FCoV genotype from type II-FCoV, CCoV or TGEV (Figure 3). Unfortunately, our method had no discriminating power for differentiating between type II-FCoV and CCoV genotypes.

### Comparison of FCoV detection rate from ascites, pleural effusion and blood in cats

Using the PCR and direct-sequencing method targeting nsp14 in the present study, we surveyed FCoV strains from clinically ill cats on which FIP was suspected but not confirmed by a complete clinico-pathological or pathological workup in veterinary hospitals in Japan. In peritoneal and pleural fluid samples, FCoV was detected in 55 of 103 (53.4%) and 14 of 45 (31.1%), respectively. All of these exhibited the type I-FCoV genotype. In a comparison of positivity rate of FCoV between peritoneal and pleural effusion samples, relative risk and odds ratio were 1.7164 and 2.5372 (95% confidence interval = 1.21-5.32, z statistic = 2.465, P = 0.0137), respectively, and we found statistically significant differences between the two.

On the other hand, positive results on nsp14 PCR from 214 blood samples, 9 cerebral fluid samples and 1 pericardial effusion sample from 224 clinically ill cats, were seen in 19 (8.9%), 0 (0%) and 1 (100%), respectively. Only one of the blood samples exhibited type II-FCoV or CCoV genotype.

### Population genetics of FCoV strains in Japan by nsp14 amino acid sequence typing (nsp14 aa ST)

Using nsp 14 amino acid sequence typing (nsp14 aa ST), which was an allelic analysis based on 135 amino acid residue of nsp14, we identified fifty-three unique STs among the 89 FCoV-positive samples in this study, and 39 whole genome-sequenced FCoV and CCoV strains registered in the NCBI database (Additional file 2) [27-29]. The assigned ST numbers of nsp14 aa ST in whole genome-sequenced strains are shown in Additional file 1.

**Figure 4:**
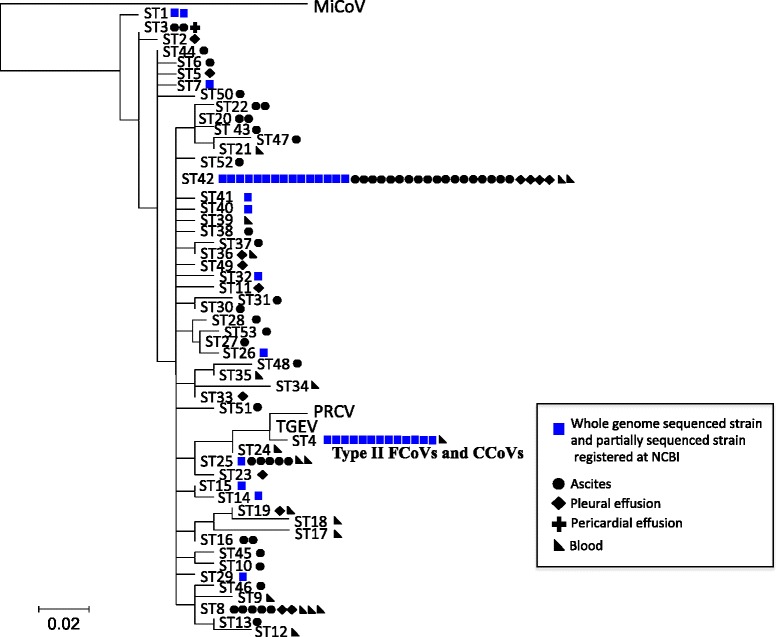
**Phylogenetic tree (ML method) based on nsp14 partial amino acid sequences (135 aa).** ST distribution and number of strains from ascites, pleural effusion, blood and pericardial effusion in population genetic structure of FCoV are indicated.

As shown in Figure 4, our nsp14 aa ST indicated that the most predominant genotype among domestic cats in Japan was ST42 (24 of 89, 27.0%), which was also found most frequently in whole genome-sequenced strains, followed by ST8 (n = 10), and ST25 (n = 7), and the top three STs accounted for 46.1% of total FCoV-positive samples. Among ascites specimens in the present study, ST42 was the most frequent genotype (n = 18), followed by ST8 (n = 5) and ST25 (n = 5). Of pleural effusion and blood samples, multiple strains of ST42 (n = 4), ST8 (n = 2) and ST8 (n = 3), ST25 (n = 2), ST42 (n = 2) were identified, respectively. We were unable to find any correlations between types of specimen and STs.

As shown in Table 1, populations of FCoV strains from blood specimens showed high values for both diversity and evenness indexes, which indicates an almost random pattern. The lowest values for both diversity and evenness indexes in ascites suggested the presence of ascites-tropic clones. In addition to FCoV detection rate, population structures of FCoV strains between ascites and pleural effusion specimens also exhibited different trends.

**Table 1:** **Diversity and evenness indexes of FCoV strains by specimens**

**Kind of specimen**	**No. of specimens**	**No. of strains**	**No. of STs**	**Simpson’s index (1-λ)**	**Pielou’s index**	**Predominant ST(s)** ^**a**^
Ascites	103	55	26	0.881	0.661	ST42
Pleural effusion	45	14	10	0.923	0.812	ST8, ST42
Blood	214	19	15	0.971	0.895	ST8, ST25, ST42

^a^ST(s) which accounted for not less than 10% of clones in the population.

## Discussion

In previous epidemiological studies, nested PCR techniques targeting 3′-UTR and spike sequences have been widely used for viral detection and discrimination between type I and II serotypes, respectively [3,12,30-36]. However, previous methods are time-consuming, and nonspecific amplification often occurs in the course of nested PCR. Our nsp14 PCR method allowed detection of FCoV using a single PCR, and little nonspecific amplification occurred during PCR in various clinical specimens, suggesting that the primer set exhibited high specificity to only viral genome. Consequently, our method allows to ascertain accurately the presence of FCoV in any type of specimen without confirmation by sequencing of PCR products. Recently, Soma et al. reported that positivity of FCoV was 44.1% (377 of 854) in ascites from cats suspected of wet FIP in Japan using the 3′-UTR nested PCR method [36]. Although a simple comparison was not clear because of different study populations, detection sensitivity of FCoV in ascites specimens of our nsp14 single PCR was comparable or better (P = 0.0746), compared with the study using the nested PCR method. There have been some reports on the quasispecies in FCoV [31,37-41]; Battilani et al. reported that quasispecies composition is correlated with the seriousness of clinical form and lesions in the organs [37]. Therefore, detection of a population that consists of a complex of heterogeneous variants is necessary in the diagnosis of FCoV infection. Consequently, this may have resulted in improved sensitivity for FCoV detection by the present PCR targeting nsp14, which is a highly conserved region among alpha-coronavirus1 subspecies. The present method is the most reliable PCR method that can tolerate the genetic diversity of FCoV.

Using the scheme of nsp14 aa ST, we found that a clone of FCoV, ST42, is prevalent in domestic cats in Japan. It is likely that the ST42 FCoV clone is also endemic worldwide, as it accounts for most of the whole genome-sequenced strains registered at NCBI. The global distribution of ST8 and ST25 FCoV clones, which are second and third most common STs after ST42 in Japan, remains to be clarified. Further epidemiological study is thus needed.

Aggregated distribution of specific STs in FCoV strains from ascites was in contrast to that of blood specimens that formed an almost random pattern, suggesting that a tropic ascites clone is present among FCoV populations. A previous study also reported differences of quasispecies compositions of FCoV in ORF7b and N region between organs [38]. Interestingly, diversity and evenness of FCoV strains from pleural effusion were similar to those of blood but not ascites. With regard to both viral detection rate and population structures, different trends between ascites and pleural effusion specimens suggest that pathology of FCoV relevant-pleural effusion or pleural FIP have markedly different characteristics from those of FCoV relevant-ascites or peritoneal FIP. Previous studies reported that genomic alteration or variability of viral population during infection could affect the organ-specificity, severity and immunological escape [37,39,41]. Although, an onset of FCoV infection or FIP highly depends on host factors, FCoV also might exhibit the pathogenic diversity by viral strains.

The so-called internal mutation hypothesis, which postulates that viruses transition from avirulent to virulent via certain mutations leading to FIP pathogenesis, is generally believed in veterinary medicine [10]. However, there have been no reports on the identification of consensus mutations in any FCoV strains; no relationship between viral phylogeny and virulence has previously been found [42-44]. Our diversity analysis did not search for mutations in the FCoV genome responsible for FIP pathogenesis, suggesting that genetically diverse FCoV clones are present in domestic cats and that dynamically selected clones can cause FCoV-related ascites or wet FIP. Other reports also suggested that sequential emergence of variants and replacing the pre-existing population occurred in FCoV under the host immune pressure [39,41]. Thus, transition to FIP may occur through changes in viral populations in a feline host rather than internal mutations.

Recently, severe acute respiratory syndrome (SARS) in 2002/2003 and Middle East respiratory syndrome (MERS) in 2012 have emerged as human infectious disease from zoonotic coronaviruses [45,46]. To trace the original infectious source of coronavirus zoonotic transmission, we need to understand the ecology and population structures of viral strains in various animal species. Although there have been reports on detection of strains with the FCoV-genotype in host species of Carnivora other than domestic cats [35,47-49], our ecological understanding of this virus remains insufficient. The present method, which was applicable to all alpha-coronavirus1 subspecies, will also contribute to our ecological understanding of this virus.

## Conclusions

In conclusion, we developed a detection and genotyping tool for all variants of FCoV, and confirmed the presence of an endemic FCoV clone, ST42, in Japan and probably worldwide. The present nsp14 PCR method will contribute to molecular epidemiology and ecological findings in alpha-coronavirus 1 subspecies, including FCoV.

## Additional files

Additional file 1:
**Coronavirus strains for whole genome analysis used in the present study.** Thirty-two FCoV strains, seven canine coronavirus (CCoV) strains, four transmissible gastroenteritis coronavirus (TGEV) strains, a porcine respiratory coronavirus (PRCV) strain and a Mink coronavirus (MiCoV) strain were used for comparative genome analysis. The assigned ST numbers of nsp14 aa ST in whole genome-sequenced strains are shown. Additional file 2:
**Amino acid sequences of each ST in nsp14 aa ST.** Amino acid sequences, representative strains and the accession numbers of fifty-three unique STs in nsp14 aa ST are shown in this table. The sequences of representative strains detected in the present study have been deposited in the DDBJ/GenBank database.

## Abbreviations

FCoV: Feline coronavirus
WGSs: Whole genome sequences
nsp14 aa ST: nsp14 amino acid sequence typing
ST: Sequence type
FECV: Feline enteric coronavirus
FIPV: Feline infectious peritonitis virus
CCoV: Canine coronavirus
TGEV: Transmissible gastroenteritis coronavirus
PRCV: Porcine respiratory coronavirus
MiCoV: Mink coronavirus
ML: Maximum likelihood
SARS: Severe acute respiratory syndrome
MERS: Middle East respiratory syndrome
M.W.M: Molecular weight marker
